# The factors related to decreases in masticatory performance and masticatory function until swallowing using gummy jelly in subjects aged 20‐79 years

**DOI:** 10.1111/joor.12975

**Published:** 2020-04-20

**Authors:** Keitaro Ohno, Yuko Fujita, Yoma Ohno, Tomohiro Takeshima, Kenshi Maki

**Affiliations:** ^1^ Division of Developmental Stomatognathic Function Science Department of Health Promotion Kyushu Dental University Kitakyushu City Japan

**Keywords:** adults, eating habit, general health, masticatory performance, swallowing threshold, tongue pressure

## Abstract

**Background:**

There is growing international interest in the prevention of decreased oral function for managing oral health in older people.

**Objective:**

The aims of the present study were to identify factors related to decreases in masticatory performance and masticatory function until swallowing in subjects aged 20‐79 years old.

**Methods:**

A total of 152 subjects, ranging in age from 20 to 79 years, were divided into six groups according to their chronological age: 20‐29, 30‐39, 40‐49, 50‐59, 60‐69 and 70‐79 years. Grip strength, maximum occlusal force, maximum tongue pressure, masticatory performance and swallowing threshold were measured in all subjects. Masticatory performance and swallowing threshold were determined according to the concentration of dissolved glucose obtained from gummy jellies; decreased masticatory performance and decreased swallowing threshold were defined as glucose concentrations in the lowest 20th percentile. A multivariate binary logistic regression analysis was used to identify factors associated with decreased masticatory performance and decreased swallowing threshold. A self‐administered lifestyle questionnaire was also completed.

**Results:**

Logistic regression analyses revealed that factors related to decreased masticatory performance included use of more than one kind of medicine for treating chronic diseases and removable denture use, while factors related to decreased swallowing threshold included eating between meals once or more per day, poorer mental health and decreased saliva flow.

**Conclusions:**

Different factors are related to decreased masticatory performance and decreased swallowing threshold, although both of these phenomena are closely associated with general health status.

## 1 INTRODUCTION

There is increasing international interest in the identification of appropriate strategies for management of age‐related decline in oral health and prevention of decreased oral function in older people.^1^ In 2016, the Japanese Society of Gerodontology presented criteria including decrease in tongue pressure, decrease in masticatory function and deterioration of swallowing function for diagnosis of oral hypofunction in older people.^1^ Recent studies have indicated that decreased tongue strength may be an important factor in nutrition‐related sarcopenia.^2^, ^3^ Moreover, several studies have reported that masticatory performance is closely related to hand grip strength in adult subjects.^4^, ^5^ These findings suggest that oral hypofunction is linked to skeletal sarcopenia or poor general health status; simultaneously, we hypothesised that decrease in masticatory function would be related to physical and mental health status as well as dental health status.

There are many studies on the factors related to the masticatory performance including the number of residual teeth and occlusal force.^4^, ^5^, ^6^ However, there have been fewer studies on factors related to the swallowing threshold than on masticatory performance; a study reported that subjects with shortened dental arches without occluding molars had higher number of chewing cycles and longer chewing time until swallowing test foods than the subjects with complete dental arches.^7^ However, few studies have evaluated masticatory performance and swallowing threshold simultaneously in adult subjects. A recent study reported that better mental health was associated with a higher swallowing threshold in subjects in their 20s.^8^ These findings suggest that some people begin swallowing foods with less chewing at a relatively young age.

Earlier countermeasures could be implemented if lifestyle factors, general health and oral health could be used to predict decreased masticatory performance and swallowing threshold, potentially preventing nutrition‐related sarcopenia.

The aims of the present study were to determine changes in various oral functions according to age, as well as factors related to decreased masticatory performance and decreased masticatory function until swallowing in 20‐79 years old.

## 2 METHODS

### 2.1 Participants

This study was approved by the Human Investigation Committee of Kyushu Dental University (Kitakyushu, Fukuoka, Japan; Approval Number 16‐27), and all subjects provided written informed consent before participation. Subjects visited one private dental clinic in Japan for an oral examination, based on which the final study participants were selected. The inclusion criteria were as follows^9^, ^10^: (a) normal language comprehension; (b) ability to independently perform activities of daily living (ADL), including feeding, sit to stand (chair to bed), personal grooming (washing, shaving and combing), getting on/off the toilet, bathing, walking on level surfaces, ascending and descending stairs, dressing and control of bowel function and bladder, based on the assessment variables in the Barthel index^11^; (c) occlusal contact of either the natural teeth or artificial teeth including removable dentures at the first molars; and (d) no current dental diseases or complications. The exclusion criteria included systemic disturbances causing swallowing impairments, obvious facial asymmetry that could affect the recordings, soft tissue abnormalities, temporomandibular joint dysfunction and irregularities in the form or structure of the natural teeth.

A previous study reported that the mean masticatory performance among adult patients was around 180 ± 30 mg/dL. If the true difference in the decreased function and normal means is 20 mg/dL,^8^ 30 decreased function participants and 120 normal function participants were calculated to be required to reject the null hypothesis that the means of the decreased and normal groups are equal with a power of 0.9. The probability of a Type I error associated with the test of this null hypothesis was 0.05. To account for potential dropouts, we recruited 152 adults aged 20‐79 years (76 males and 76 females).

The subjects were divided into six groups according to their chronological age, with each group being further divided into two subgroups according to gender (20s, 12 males and 15 females; 30s, 12 males and 13 females; 40s, 15 males and 12 females; 50s, 13 males and 12 females; 60s, 12 males and 12 females; and 70s, 12 males and 12 females).

### 2.2 Anthropometry and dental examination

Height and body weight were measured in the consultation room of the clinic. Height was measured to an accuracy of ±0.1 cm using a portable digital stadiometer (AD‐653; A&D), with the head in the Frankfort plane, while body weight was measured to an accuracy of 0.1 kg.^12^

During the intra‐oral examination, the number of functional teeth and total number of decayed, missing and filled teeth (DMFT) were recorded, and the Community Periodontal Index (CPI) was recorded according to the criteria recommended by the World Health Organization.^13^

### 2.3 Questionnaire

The survey solicited the following information: demographic characteristics (sex and age), eating habits, physical activity, sleep, current and past smoking habits, alcohol use, removable denture use and use of medicines to treat for chronic diseases (including hypertension, arrhythmia, diabetes, hypercholesterolaemia, anaemia and others). Mental health was assessed using the 12‐item General Health Questionnaire (GHQ‐12; Japanese version). A higher score indicated poorer mental health status. The validity and reliability of the GHQ‐12 have been confirmed in a previous study.^14^

### 2.4 Hand grip strength

Hand grip strength was measured using a portable grip strength meter (T‐2288; TOEI LIGHT Co. Ltd.). Participants were asked to stand and hold a dynamometer in their hand with their arm parallel to the body, without squeezing the arm against the body. Hand grip strength was measured, in kg, twice for each hand (alternately) with a 30‐s interval between trials. The highest value from either the left or right hand was recorded as the grip strength.^8^

### 2.5 Maximum occlusal force

Maximum occlusal force was measured using a portable occlusal force meter (GM10; Nagano Keiki Co. Ltd.), which consisted of a strain gauge in the centre of a biting element encased in a plastic tube. Participants were examined while relaxed in a sitting position, with the Frankfort plane horizontal. Participants were asked to place the element on the maxillary first molar or second primary molar and bite it with maximal voluntary muscular effort for approximately 3 s. Occlusal force was measured in kilonewtons (kN) by a digital pressure gauge built into the element. The maximum bite force was measured on each side with a 30‐s interval between bite measurements. The larger of the values recorded on the left and right sides was considered to be the maximum bite force and was used in subsequent analyses.^8^, ^9^

### 2.6 Maximum tongue pressure

Maximum tongue pressure was measured using a tongue pressure manometer (JMS). Participants were examined while relaxed in a sitting position and were asked to place a balloon on the anterior part of their palate and close their lips, biting a hard ring with the upper and lower incisors. Then, they were asked to raise their tongues and compress the balloon onto the palate with maximal voluntary muscular effort for approximately 7 s. The pressure was measured (in kilopascals) using a digital voltmeter attached to the tongue pressure manometer.^8^, ^9^

### 2.7 Masticatory performance

Masticatory performance was assessed according to the concentration of dissolved glucose obtained from a cylindrical‐shaped gummy jelly consisting of 40% maltose, 10% sorbitol and 5% glucose (GLUCOLUMN; GC Co. Ltd.). Before the experiments, participants were shown how to perform the masticatory movements and mouth‐rinsing procedure to ensure that they would not swallow. The participants were then instructed to chew the gummy jelly on their habitual chewing side (left, right or both) for 20s. After chewing, the participants were asked to take 10 mL of distilled water into their mouth and to spit out the gummy jelly, distilled water and saliva into a filter cup. The glucose concentration in the filtrate (mg/dL) was measured using a reliable, previously validated glucose‐measuring device (GLUCO SENSOR GS‐II; GC Co. Ltd.) to measure masticatory performance.^8^ A glucose concentration in the lowest 20th percentile was defined as decreased masticatory performance based on the previous studies regarding the criteria of sarcopenia.^15^, ^16^, ^17^

### 2.8 Swallowing threshold and stimulated salivary flow

Following evaluation of masticatory performance, an assessment of swallowing threshold was performed using the gummy jellies (GLUCOLUMN, GC Co. Ltd.). The three variables related to swallowing (ie number of chewing cycles, chewing time and glucose concentration in the filtrate [ml/dl]), were assessed in all participants. In the studies on dysphagia, "decreased swallowing threshold" means more sensitive to stimuli for swallowing.^18^, ^19^ In this study, we defined "decreased swallowing threshold" as the condition that food is swallowed with less chewing, and used the glucose concentration obtained from chewed gummy jelly as the swallowing threshold just before the participants determined that swallowing was possible. Each participant was instructed to chew a gummy jelly on their habitual chewing side. Participants were then instructed to chew until feeling the desire to swallow, at which time they were instructed to stop chewing and signal to the examiner that they were ready to expel the gummy jelly. The examiner counted the number of chewing cycles, and the time from the onset of chewing to the moment at which participants raised their hand was recorded using a stopwatch. The subsequent steps were the same as those used for the evaluation of masticatory performance.^8^

Whole saliva was collected to evaluate the swallowing threshold. All filtrates with gummy jelly pieces removed were used to measure the stimulated salivary flow, with the amount thereof calculated by deducting 10 mL from the total amount of filtrate. All saliva specimens were collected between 10:00 am and 3:00 pm ^6^ A glucose concentration in the lowest 20th percentile was defined as decreased swallowing threshold based on previous studies regarding the criteria of sarcopenia.^15^, ^16^, ^17^

### 2.9 Reliability of measurements

All measurements were performed in duplicate, separated by a 30‐s rest period, and the mean values were used in subsequent analyses. All examinations were performed by the same examiner. The data were assessed in terms of intra‐rater reliability using the intra‐class correlation coefficient (ICC; 0.800 ≤ ICC ≤ 1.000 corresponds to high reliability).^20^

### 2.10 Data analysis

The Shapiro‐Wilk test was used to determine the normality of the data. All continuous data are expressed as mean ± standard deviation (SD). Mean values were compared between the two groups using a two‐tailed *t* test or the Mann‐Whitney *U* test. The Kruskal‐Wallis test was used for comparison of more than two groups. The chi‐squared test or Fisher's exact test was used as appropriate to compare the normal masticatory performance and decreased masticatory performance (lowest 20%) groups, and the normal swallowing threshold and decreased swallowing threshold (lowest 20%) groups, in terms of categorical variables. Binary logistic regression analysis with the forward selection (conditional) method was used to identify factors predicting decreased masticatory performance and decreased swallowing threshold in each group. Independent variables that were significant in the univariate analyses were included. Categorical variables were coded appropriately before being entered into the model. The adjusted odds ratios (ORs), and their 95% confidence intervals (CIs), were calculated for the low masticatory performance and low swallowing threshold groups. A *P*‐value <.05 was considered to indicate statistical significance. All data were analysed using SPSS for Windows software (version 23.0; IBM Japan).

## 3 RESULTS

The ICCs for height, body weight, number of functional teeth, DMFT index, CPI, GHQ‐12 score, hand grip strength, maximum occlusal force, maximum tongue pressure, masticatory performance, number of chewing cycles, chewing time, glucose concentration and stimulated salivary flow until swallowing were all ≥0.83.

The anthropometric, dental examination and GHQ‐12 data are shown in Table 1 by age and sex. The number of functional teeth in 70‐ to 79‐year‐old males and females was significantly lower than that in the males and females in the other groups (all, *P* < .05). The DMFT index of females in their 70s was significantly lower than that of males and females in their 20s and 30s (all, *P* < .05).

**TABLE 1:** Anthropometric parameters, dental examination results and GHQ‐12 score by age group and sex

Age group	Sex (n = 152)	Age (y)	Height (m)	Body weight (kg)	Body mass index (kg/m^2^)	Number of functional teeth	DMFT index	Community Periodontal Index	GHQ‐12 score
20s	M (n = 12)	23.67 ± 3.52	1.66 ± 0.07^‡^	61.54 ± 7.18	22.55 ± 3.35	26.25 ± 1.91^†^ ^,^ ^‡^	4.00 ± 4.09^†^ ^,^ ^‡^	2.25 ± 0.97	2.92 ± 1.88
	F (n = 15)	24.53 ± 2.53	1.56 ± 0.05*	46.47 ± 4.03* ^,^ ^†^	19.05 ± 1.49*	27.93 ± 0.26* ^,^ ^†^ ^,^ ^‡^	6.20 ± 4.18^†^ ^,^ ^‡^	1.60 ± 0.74^†^ ^,^ ^‡^	2.07 ± 1.10
30s	M (n = 12)	33.92 ± 3.78	1.68 ± 0.03^‡^	66.17 ± 6.03	23.33 ± 2.02	27.42 ± 1.38^†^ ^,^ ^‡^	13.17 ± 5.18^‡^	2.75 ± 0.87	2.00 ± 2.22
	F (n = 13)	35.46 ± 2.79	1.56 ± 0.06*	48.62 ± 6.86* ^,^ ^†^	19.90 ± 2.76*	27.46 ± 0.78^†^ ^,^ ^‡^	12.38 ± 5.41^‡^	2.69 ± 0.85	2.15 ± 1.46
40s	M (n = 15)	46.13 ± 2.17	1.68 ± 0.05^‡^	73.00 ± 13.55^‡^	25.91 ± 4.50	26.60 ± 1.72^†^ ^,^ ^‡^	16.00 ± 5.44	2.93 ± 0.59	1.07 ± 1.03
	F (n = 12)	44.58 ± 2.78	1.60 ± 0.05* ^,^ ^‡^	53.50 ± 6.61*	20.88 ± 2.06*	26.75 ± 1.76^†^ ^,^ ^‡^	17.58 ± 7.28	2.75 ± 0.87	1.83 ± 2.21
50s	M (n = 13)	54.15 ± 2.91	1.68 ± 0.08^‡^	65.46 ± 7.99	23.29 ± 2.21	26.08 ± 2.10^†^ ^,^ ^‡^	14.62 ± 4.61	2.85 ± 0.80	1.77 ± 1.24
	F (n = 12)	55.08 ± 2.15	1.54 ± 0.03* ^,^ ^†^	53.83 ± 8.99*	22.59 ± 3.46	26.50 ± 1.17^†^ ^,^ ^‡^	18.83 ± 5.27*	3.00 ± 0.95	1.83 ± 1.19
60s	M (n = 12)	63.25 ± 2.22	1.71 ± 0.06	73.58 ± 13.02^‡^	25.19 ± 3.41	24.42 ± 2.57^†^ ^,^ ^‡^	19.92 ± 3.09	3.42 ± 0.51	2.25 ± 1.42
	F (n = 12)	65.50 ± 2.54	1.54 ± 0.04*	52.75 ± 5.58*	22.15 ± 2.09*	24.33 ± 2.64^†^ ^,^ ^‡^	17.25 ± 4.20	2.83 ± 0.58*	1.17 ± 0.72*
70s	M (n = 12)	74.92 ± 3.03	1.65 ± 0.06	63.92 ± 7.19	23.42 ± 1.75	18.92 ± 5.23	20.58 ± 3.48	3.50 ± 0.67	2.00 ± 1.35
	F (n = 12)	73.75 ± 2.80	1.50 ± 0.06* ^,^ ^†^	52.67 ± 7.94*	23.39 ± 2.50	17.83 ± 6.83	23.25 ± 4.65	3.17 ± 0.72	1.42 ± 1.16

Note

Data are expressed as mean ± standard deviation. Differences between males and females within each group were assessed by a two‐tailed *t* test or the Mann‐Whitney *U* test. Differences between males or females in their 70s and other groups were assessed by a Kruskal‐Wallis test.

Abbreviations: DMFT, decayed, missing and filled teeth; F, female; GHQ, General Health Questionnaire; M, male.

* *P* < .05 vs group‐matched males.

^†^ *P* < .05 vs 70s males.

^‡^ *P* < .05 vs 70s females.

**FIGURE 1:**
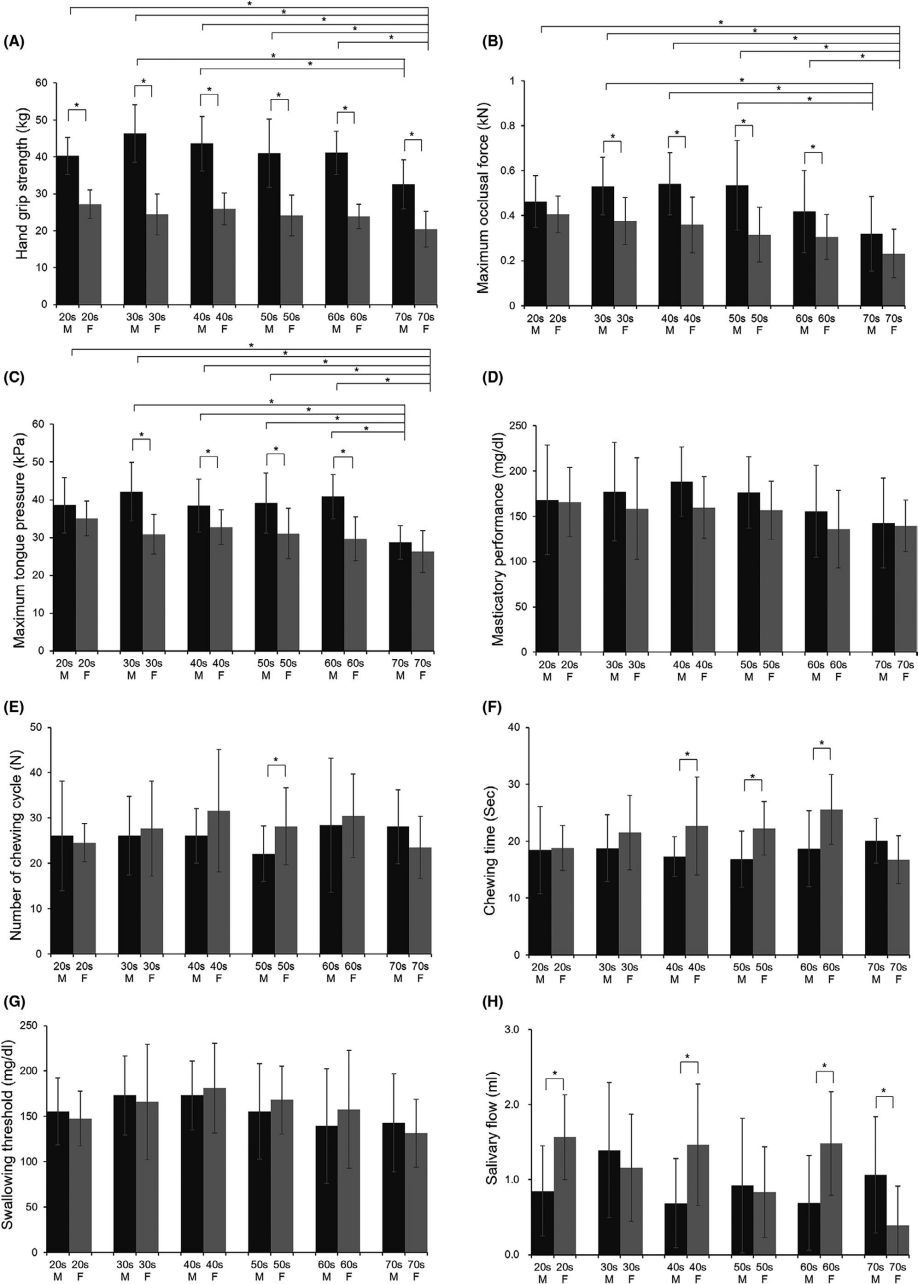
Comparison of hand grip strength, oral functions and stimulated salivary flow by age group and sex. Data are expressed as mean ± standard deviation. M, male; F, female. (A) Hand grip strength; (B) maximum occlusal force; (C) maximum tongue pressure; (D) masticatory performance; (E) number of chewing cycle; (F) chewing time; (G) swallowing threshold; (H) stimulated salivary flow. Differences between males and females within each group were assessed by a two‐tailed *t* test or the Mann‐Whitney *U* test. Differences between males or females in their 70s and other groups were assessed by a Kruskal‐Wallis test. *P < .05

The mean scores for hand grip strength, maximum occlusal force, maximum tongue pressure, masticatory performance and swallowing threshold are shown in Figure 1. Maximum occlusal force and maximum tongue pressure were significantly higher for males than females within four groups (30s, 40s, 50s and 60s; all *P* < .05). No significant difference in masticatory performance was seen by age or sex. Among males, the mean hand grip strength and maximum tongue pressure were highest in those in their 30s vs all other age groups, and the difference between the 30s and 70s age groups was significant (all, *P* < .05). Among females, the mean hand grip strength, maximum occlusal force and maximum tongue pressure were highest in those in their 20s vs all other age groups. The glucose concentration until swallowing did not differ by age or sex.

Table 2 summarises the data on decreased masticatory performance and decreased swallowing threshold by demographic and health‐related variables. Participants with decreased masticatory performance were more likely to be older, to be former smokers, to take more than one medicine for chronic diseases and to use removable dentures *(P* = .005, *P* = .001, *P* < .0005 and *P* < .0005, respectively).

**TABLE 2:** Decreased masticatory performance and decreased swallowing threshold according to demographic and health‐related variables

Participants (n = 152)	Normal masticatory performance (%)	Decreased masticatory performance (%)	*χ* ^2^	*P*‐value	Normal swallowing threshold (%)	Decreased swallowing threshold (%)	*χ* ^2^	*P*‐value
Gender								
Female	60 (49.6)	16 (51.6)			61 (50.0)	15 (50.0)		
Male	61 (50.4)	15 (49.4)			61 (50.0)	15 (50.0)		
			—	1.000^†^			—	1.000^†^
Age (y)								
20‐29	24 (19.8)	3 (9.7)			23 (18.9)	4 (13.3)		
30‐39	21 (17.4)	4 (12.9)			20 (16.4)	5 (16.7)		
40‐49	25 (20.7)	2 (6.5)			25 (20.5)	2 (6.7)		
50‐59	22 (18.2)	3 (9.7)			22 (18.0)	3 (10.0)		
60‐69	15 (12.4)	9 (29.0)			18 (14.8)	6 (20.0)		
70‐79	14 (11.6)	10 (32.3)			14 (11.5)	10 (33.3)		
			16.635	.005^‡^			11.655	.040^‡^
Eating between meals								
Less than once a day	49 (40.5)	13 (41.9)			55 (45.1)	7 (23.3)		
Once or more a day	72 (59.5)	18 (58.1)			67 (54.9)	23 (76.7)		
			0.021	.884^‡^			—	.038^†^
Physical activity								
30 min or more a day	30 (24.8)	4 (12.9)			30 (24.6)	4 (13.3)		
Less than 30 min a day	20 (16.5)	5 (16.1)			25 (20.5)	0 (0.0)		
None	71 (58.7)	22 (71.0)			67 (54.9)	26 (86.7)		
			2.171	.338^‡^			11.478	.003^‡^
Self‐assessed sleep quality								
Good	79 (65.3)	22 (71.0)			84 (68.9)	17 (56.7)		
Poor	42 (34.7)	9 (29.0)			38 (31.1)	13 (43.3)		
			0.357	.550^‡^			—	.280^†^
Alcohol drinking								
Never	53 (43.8)	19 (61.3)			56 (45.9)	16 (53.3)		
Less than three times a week	42 (34.7)	8 (25.8)			41 (34.4)	8 (26.7)		
Three times or more a week	26 (21.5)	4 (12.9)			24 (19.7)	6 (20.0)		
			2.998	.211^‡^			0.637	.727^†^
Smoking								
Never	94 (79.0)	19 (61.3)			93 (76.9)	20 (69.0)		
Former	5 (4.2)	8 (25.8)			9 (7.4)	4 (13.8)		
Current	20 (16.8)	4 (12.9)			19 (15.7)	5 (17.2)		
			14.503	.001^‡^			1.318	.517^‡^
Medicines for chronic diseases								
0	84 (69.4)	9 (29.0)			76 (62.3)	17 (56.7)		
1	34 (28.1)	19 (61.3)			41 (33.6)	12 (40.0)		
≥2	3 (2.5)	3 (9.7)			5 (4.1)	1 (3.3)		
			17.615	<.0005^‡^			0.443	.801^‡^
Use of removable dentures								
No	114 (94.2)	17 (54.8)			108 (88.5)	23 (76.7)		
Yes	7 (5.8)	14 (45.2)			14 (11.5)	7 (23.3)		
			—	<.0005^†^			—	.135^†^

^†^ Fisher's exact test.

^‡^ chi‐squared test.

Participants with a decreased swallowing threshold were more likely to be older, eat between meals once or more per day and have a low physical activity level (*P* = .040, *P* = .038 and *P* = .003, respectively). Of the participants in this study, 28 took medicine for hypertension, 16 took medicine for arrhythmia, and 5 took medicine for diabetes or hypercholesterolaemia at fixed intervals (data not shown). Additionally, as the results of Kruskal‐Wallis test, there were no significant differences in stimulated salivary flow according to the number of regular medicines (data not shown).

Table 3 summarises the data on decreased masticatory performance and decreased swallowing threshold by age, body weight, dental and mental health status, and muscle strength. Regarding decreased masticatory performance, the number of functional teeth, CPI, hand grip strength, maximum occlusal force, maximum tongue pressure and swallowing threshold in the decreased masticatory performance group were significantly lower than those in the normal masticatory performance group (all, *P* < .05). The mean age and mean DMFT index in the decreased masticatory performance group were significantly higher than those in the normal masticatory performance group (both *P* < .05).

**TABLE 3:** Decreased masticatory performance and decreased swallowing threshold according to age, body weight, dental and mental health status, and muscle strength

	Normal masticatory performance (n = 121)	Decreased masticatory performance (n = 31)	Normal swallowing threshold (n = 122)	Decreased swallowing threshold (n = 30)
Age (y)	46.65 ± 16.22	57.94 ± 18.66*	47.56 ± 16.42	54.63 ± 19.75^†^
Height (m)	1.62 ± 0.08	1.59 ± 0.09	1.62 ± 0.08	1.60 ± 0.10
Body weight (kg)	59.71 ± 12.72	57.58 ± 9.67	59.59 ± 12.18	58.03 ± 12.23
Body mass index (kg/m^2^)	22.61 ± 3.50	22.64 ± 2.68	22.62 ± 3.40	22.59 ± 3.13
Number of functional teeth (N)	26.00 ± 3.20	21.84 ± 5.79*	25.73 ± 3.44	22.80 ± 5.92^†^
DMFT index	14.38 ± 7.23	18.03 ± 6.48*	14.53 ± 7.00	17.53 ± 7.68^†^
Community Periodontal Index	2.71 ± 0.94	3.10 ± 0.60*	2.74 ± 0.88	3.00 ± 0.95
GHQ‐12 score (point)	1.88 ± 1.59	1.77 ± 1.06	1.65 ± 1.48	2.73 ± 1.23^†^
Hand grip strength (kg)	33.83 ± 10.90	28.23 ± 8.21*	33.42 ± 10.68	29.71 ± 10.02
Maximum occlusal force (kN)	0.42 ± 0.15	0.32 ± 0.17*	0.42 ± 0.15	0.34 ± 0.18^†^
Maximum tongue pressure (kPa)	35.64 ± 7.71	30.36 ± 6.48*	35.03 ± 7.47	32.65 ± 8.71
Masticatory performance (mg/dL)	177.03 ± 36.22	98.71 ± 13.66*	170.87 ± 43.16	121.17 ± 31.90^†^
Number of chewing cycle (N)	26.90 ± 10.01	26.45 ± 6.79	28.34 ± 9.40	20.57 ± 6.58^†^
Chewing time (s)	19.41 ± 6.17	21.00 ± 5.55	20.89 ± 5.80	15.03 ± 4.75^†^
Swallowing threshold (mg/dL)	167.46 ± 47.09	119.55 ± 36.69*	173.53 ± 40.98	93.27 ± 12.85^†^
Salivary flow (mL)	1.08 ± 0.80	0.92 ± 0.63	1.14 ± 0.76	0.66 ± 0.67^†^

Note

Data are expressed as mean ± standard deviation. Differences between normal and low groups were assessed by a two‐tailed *t* test or the Mann‐Whitney *U* test.

Abbreviations: DMFT, decayed, missing and filled teeth; GHQ, General Health Questionnaire; Swallowing threshold, glucose concentration on first swallow.

* *P* < .05 vs normal masticatory performance group.

^†^ *P* < .05 vs normal swallowing threshold group.

Regarding decreased swallowing threshold, the number of functional teeth, maximum occlusal force, masticatory performance, number of chewing cycles, chewing time and salivary flow in the decreased swallowing threshold group were significantly lower than those in the normal swallowing threshold group (all, *P* < .05). The mean age, mean DMFT index and GHQ‐12 scores in the decreased swallowing threshold group were significantly higher than those in the normal swallowing threshold group (all, *P* < .05).

Table 4 shows the predictors of decreased masticatory performance, as revealed by logistic regression. The use of more than one medicine for treating chronic diseases (OR = 10.919, *P* = .008, 95% CI = 1.891‐63.053) and use of removable dentures (OR = 10.198, *P* < .0005, 95% CI = 3.215‐32.354) were both associated with higher odds of decreased masticatory performance.

**TABLE 4:** Predictors of decreased masticatory performance based on logistic regression analysis

Independent variables	Category	Adjusted odds ratio (95% CI)	*P*‐value	Score assigned
Number of regular medicines for chronic diseases	0	1	—	0
	1	2.466 (0.880‐6.915)	.086	1
	≥2	10.919 (1.891‐63.053)	.008	2
Use of removable dentures	No	1	—	0
	Yes	10.198 (3.215‐32.354)	<.0005	1

Note

Forward selection (conditional) method. −2 Log likelihood = 119.867. Hosmer and Lemeshow test: *χ* ^2^ = 0.210, *P* = .900. Cox‐Snell *R* ^2^ = .200. Nagelkerke *R* ^2^ = .314.

Abbreviation: CI, confidence interval.

Table 5 shows the predictors of decreased swallowing threshold, as revealed by logistic regression. Eating between meals once or more a day (OR = 3.390, *P* = .021, 95% CI = 1.198‐9.591) was associated with higher odds of a decreased swallowing threshold. For every point increase in the GHQ‐12 score, the odds of a decreased swallowing threshold increased by a factor of 1.566 (*P* = .001, 95% CI = 1.165‐2.104). In contrast, for every 0.1 mL increase in the salivary flow before swallowing, the odds of a decreased swallowing threshold dropped by a factor of 0.282 (*P* = .004, 95% CI = 0.137‐0.582).

**TABLE 5:** Predictors of decreased swallowing threshold based on logistic regression analysis

Independent variables	Category	Adjusted odds ratio (95% CI)	*P*‐value	Score assigned
Eating between meals	Less than once a day	1	—	0
	Once or more a day	3.390 (1.198‐9.591)	.021	1
GHQ‐12 score (per 1‐point increase)		1.566 (1.165‐2.104)	.001	—
Salivary flow (per 0.1‐ml increase)		0.282 (0.137‐0.582)	.004	—

Note

Forward selection (conditional) method. −2 Log likelihood = 121.441. Hosmer and Lemeshow test: *χ* ^2^ = 9.239, *P* = .323. Cox‐Snell *R* ^2^ = .177. Nagelkerke *R* ^2^ = .281.

Abbreviations: CI, confidence interval; GHQ, General Health Questionnaire.

## 4 DISCUSSION

In the present study, consistent differences in hand grip strength and maximum tongue pressure were observed by age group. These results are in agreement with previous studies.^21^, ^22^ Maximum tongue pressure significantly declined in males in their 70s, whereas previous studies showed that maximum tongue pressure significantly declined in the 60s.^21^, ^23^ The reason for this difference was that, in our study, the mean height and body weight were highest in males in their 60s among all age groups, where a recent study reported that maximum tongue pressure was significantly associated with height and body weight in young adults.^9^ In females, skeletal and oral muscle strength tended to decline earlier than in males; however, the speed of decline tended to be slower than in males.

Additionally, we found that masticatory performance and swallowing threshold did not obviously differ by sex or age, unlike maximum occlusal force and maximum tongue pressure. It may be that masticatory muscle function declines more slowly because of various factors, the presence of which might also have been associated with the ability of the elderly participants in our study to independently perform ADL.

In the logistic regression analysis, the use of more than one medicine for treating chronic diseases was significantly associated with decreased masticatory performance. Previous epidemiological studies reported that periodontitis and the number of lost teeth were positively related to the prevalence of chronic diseases, including hypercholesterolaemia, hypertension and diabetes in adults,^24^, ^25^ as well as the incidence of cardiovascular disease and coronary heart disease in middle‐aged males.^26^ In the present study, the number of functional teeth in the decreased masticatory performance group was significantly lower than that in the normal masticatory performance group, and the CPI in the decreased masticatory performance group was significantly higher than that in the normal masticatory performance group, although the differences were not statistically significant in the logistic regression analysis. These findings suggest that poor tooth and periodontal health could be associated with the use of more than one medicine for chronic diseases.

In addition, the logistic regression analysis revealed that removable denture use was closely correlated with decreased masticatory performance. A previous study reported that maximum occlusal force was higher in individuals with implant fixed dental prosthesis than in those with removable dental prostheses.^27^

In the present study, removable denture use was not significantly correlated with decreased swallowing threshold. These results suggest that longer chewing time and a larger number of chewing cycles until swallowing compensated for the reduction in masticatory performance associated with use of removable dentures. Therefore, we believe that removable denture use could be less likely to be associated with decreased swallowing threshold.

The logistic regression analysis showed that eating between meals once or more per day was significantly associated with a decreased swallowing threshold. Generally, when chewing food, sensation is transmitted from the baroreceptors in the periodontal ligament and the muscle spindles in the masseter muscle to the trigeminal mesencephalic nucleus (Me5). It has been reported that the histamine nervous system receives projections from Me5, which activates the histamine nervous system and suppresses appetite via the satiety centre in animals.^28^, ^29^, ^30^ These findings suggest that individuals swallowing foods without chewing might not feel full or be able to obtain a feeling of satiety; we believe that this could explain the significantly higher prevalence of decreased swallowing threshold among those who reported eating between meals once or more a day in this study.

In addition, the logistic regression analysis showed that GHQ‐12 scores were closely correlated with a decreased swallowing threshold. A previous study indicated that GHQ‐12 scores can reflect anxiety, depression and social dysfunction.^31^ The present results were consistent with recent findings among 20‐ to 29‐year‐old young adults.^8^ Additionally, another study reported a higher chewing frequency per second after stress induction compared to resting conditions in adult subjects.^32^ These findings suggest that a decreased swallowing threshold may be related to anxiety, depression or personality characteristics rendering an individual more vulnerable to stress. In the present study, the causal relationship could not be revealed, but another possibility is that feeding behaviours such as swallowing foods with less chewing may contribute to poor mental health status.

In this study, stimulation of salivary flow until swallowing was also significantly associated with a decreased swallowing threshold. In previous studies, age seemed to play an important role in unstimulated salivary flow rates,^33^ while stimulated saliva secretion rates were not significantly different between healthy old and young adults.^34^, ^35^ In the present study, there were no significant differences in stimulated salivary flow according to age or the number of regular medicines. We suggest that the reduced salivation of individuals with a decreased swallowing threshold was due to fewer chewing cycles and a shorter chewing time.

Thus, the factors related to decreased masticatory performance and decreased swallowing threshold were closely associated with not only oral health status but also general health status in subjects aged 20‐79 years.

Our results showed that 9 out of 30 individuals with a decreased swallowing threshold were aged below 40 years. It could be suggested that the habits of eating quickly and swallowing with less chewing, beginning at a young age, can cause choking and coughing fits, a decline in swallowing muscle strength and function, and even an increased risk of death from suffocation and aspiration. It is a natural providence that muscular strength declines with ageing; however, we believe that decrease in swallowing threshold will be able to prevent by chewing foods sufficiently before swallowing and keeping good physical and mental health from young adulthood.

The present study had several limitations. First, the number of participants was small compared to previous epidemiological studies. Among the patients who visited one dental clinic, the number of patients who met all of the conditions we set was less likely as they became older. Therefore, this study was performed with the minimum required number of participants calculated by the power analysis. It may be that differences in oral function by age will be clearer in studies with higher numbers of participants. Second, this study used a cross‐sectional design, which precluded the determination of causal relationships between decreased masticatory performance and swallowing threshold with the variables included in the logistic regression analyses. Longitudinal studies are needed to investigate the relative influence of factors associated with decreased masticatory performance and decreased swallowing threshold. Third, the questionnaire section on frequency of eating between meals did not capture the number or types of snacks, so we could not discuss the association between eating between meals and decreased masticatory performance in detail. Future studies should confirm the relationship between eating between meals and decreased masticatory performance.

## 5 CONCLUSIONS

In the present study, masticatory performance and swallowing threshold did not clearly differ by sex or age, unlike maximum occlusal force and maximum tongue pressure. The factors related to decreased masticatory performance included use of more than one medicine for treating chronic diseases, and factors related to decreased swallowing threshold included poorer mental health state. Different factors were related to decreased masticatory performance and decreased swallowing threshold, although both of these phenomena were closely associated with general health status in subjects aged 20‐79 years.

## CONFLICT OF INTEREST

The authors have no financial conflict interests to disclose.

## AUTHOR CONTRIBUTIONS

KO and YF formulated the study design and drafted the whole manuscript. YO and TT analysed the data. KM supervised the data analysis. All authors read and approved the final manuscript.

## References

[1] Minakuchi S , Tsuga K , Ikebe K , et al. Oral hypofunction in the older population: position paper of the Japanese Society of Gerodontology in 2016. Gerodontology. 2018;35:317‐324. 29882364 10.1111/ger.12347

[2] Sakai K , Nakayama E , Tohara H , et al. Relationship between tongue strength, lip strength, and nutrition‐related sarcopenia in older rehabilitation inpatients: a cross‐sectional study. Clin Interv Aging. 2017;12:1207‐1214. 28814847 10.2147/CIA.S141148 PMC5546916

[3] Maeda K , Akagi J . Decreased tongue pressure is associated with sarcopenia and sarcopenic dysphagia in the elderly. Dysphagia. 2015;30:80‐87. 25248988 10.1007/s00455-014-9577-y

[4] Mihara Y , Matsuda KI , Ikebe K , et al. Association of handgrip strength with various oral functions in 82‐ to 84‐year‐old community‐dwelling Japanese. Gerodontology. 2018;35:214‐220. 10.1111/ger.12341 29766545

[5] Morita K , Tsuka H , Kato K , et al. Factors related to masticatory performance in healthy elderly individuals. J Prosthodont Res. 2018;62:432‐435. 29706464 10.1016/j.jpor.2018.03.007

[6] Ikebe K , Matsuda K , Kagawa R , et al. Association of masticatory performance with age, gender, number of teeth, occlusal force and salivary flow in Japanese older adults: is ageing a risk factor for masticatory dysfunction? Arch Oral Biol. 2011;56:991‐996. 21529776 10.1016/j.archoralbio.2011.03.019

[7] Kreulen CM , Witter DJ , Tekamp FA , Slagter AP , Creugers NH . Swallowing threshold parameters of subjects with shortened dental arches. J Dent. 2012;40:639‐643. 22521703 10.1016/j.jdent.2012.04.009

[8] Takeshima T , Fujita Y , Maki K . Factors associated with masticatory performance and swallowing threshold according to dental formula development. Arch Oral Biol. 2019;99:51‐57. 30611024 10.1016/j.archoralbio.2018.12.012

[9] Hara K , Tohara H , Kenichiro K , et al. Association between tongue muscle strength and masticatory muscle strength. J Oral Rehabil. 2019;46:134‐139. 30353915 10.1111/joor.12737

[10] Fujita Y , Ichikawa M , Hamaguchi A , Maki K . Comparison of masticatory performance and tongue pressure between children and young adults. Clin Exp Dent Res. 2018;4:52‐58. 29744216 10.1002/cre2.104 PMC5893476

[11] Mahoney FI , Barthel DW . Functional evaluation: the Barthel Index. Md State Med J. 1965;14:61‐65. 14258950

[12] Ichikawa M , Fujita Y , Hamaguchi A , Chaweewannakorn W , Maki K . Association of tongue pressure with masticatory performance and dental conditions in Japanese children. Ped Dent J. 2016;26:51‐59.

[13] World Health Organization . Oral Health Surveys: Basic Methods. 5th ed. Geneva: WHO Press; 2013 29‐56 pp. http://apps.who.int/iris/bitstream/10665/97035/1/9789241548649_eng.pdf?ua=1. Accessed December 15, 2019.

[14] Doi Y , Minowa M . Factor structure of the 12‐item General Health Questionnaire in the Japanese general adult population. Psychiatry Clin Neurosci. 2003;57:379‐383. 12839518 10.1046/j.1440-1819.2003.01135.x

[15] Fried LP , Tangen CM , Walston J , et al. Frailty in older adults: evidence for a phenotype. J Gerontol A Biol Sci Med Sci. 2001;56:M146‐156. 11253156 10.1093/gerona/56.3.m146

[16] Delmonico MJ , Harris TB , Lee JS , et al. Alternative definitions of sarcopenia, lower extremity performance, and functional impairment with aging in older men and women. J Am Geriatr Soc. 2007;55:769‐774. 17493199 10.1111/j.1532-5415.2007.01140.x

[17] Newman AB , Kupelian V , Visser M , et al. Sarcopenia: alternative definitions and associations with lower extremity function. J Am Geriatr Soc. 2003;51:1602‐1609. 14687390 10.1046/j.1532-5415.2003.51534.x

[18] Furuta T , Takemura M , Tsujita J , Oku Y . Interferential electric stimulation applied to the neck increases swallowing frequency. Dysphagia. 2012;27:94‐100. 21607745 10.1007/s00455-011-9344-2

[19] Daniels SK , Schroeder MF , McClain M , et al. Dysphagia in stroke: development of a standard method to examine swallowing recovery. J Rehabil Res Dev. 2006;43:347‐356. 17041820 10.1682/jrrd.2005.01.0024

[20] Domholdt E . Physical Therapy Research: Principles and Applications. Philadelphia: W.B Saunders Co.; 1993.

[21] Hara K , Tohara H , Kobayashi K , et al. Age‐related declines in the swallowing muscle strength of men and women aged 20‐89 years: a cross‐sectional study on tongue pressure and jaw‐opening force in 980 subjects. Arch Gerontol Geriatr. 2018;78:64‐70. 29902686 10.1016/j.archger.2018.05.015

[22] Buehring B , Hind J , Fidler E , Krueger D , Binkley N , Robbins J . Tongue strength is associated with jumping mechanography performance and handgrip strength but not with classic functional tests in older adults. J Am Geriatr Soc. 2013;61:418‐422. 23379330 10.1111/jgs.12124

[23] Utanohara Y , Hayashi R , Yoshikawa M , Yoshida M , Tsuga K , Akagawa Y . Standard values of maximum tongue pressure taken using newly developed disposable tongue pressure measurement device. Dysphagia. 2008;23:286‐290. 18574632 10.1007/s00455-007-9142-z

[24] Bronner LL , Kanter DS , Manson JE . Primary prevention of stroke. N Engl J Med. 1995;333:1392‐1400. 7477121 10.1056/NEJM199511233332106

[25] Choe H , Kim YH , Park JW , Kim SY , Lee SY , Jee SH . Tooth loss, hypertension and risk for stroke in a Korean population. Atherosclerosis. 2009;203:550‐556. 19013571 10.1016/j.atherosclerosis.2008.07.017

[26] Heitmann BL , Gamborg M . Remaining teeth, cardiovascular morbidity and death among adult Danes. Prev Med. 2008;47:156‐160. 18534671 10.1016/j.ypmed.2008.04.007

[27] Goncalves TM , Campos CH , Goncalves GM , de Moraes M , Rodrigues Garcia RC . Mastication improvement after partial implant‐supported prosthesis use. J Dent Res. 2013;92:189S‐194S. 24158344 10.1177/0022034513508556 PMC3860066

[28] Itateyama E , Chiba S , Sakata T , Yoshimatsu H . Hypothalamic neuronal histamine in genetically obese animals: its implication of leptin action in the brain. Exp Biol Med (Maywood). 2003;228:1132‐1137. 14610251 10.1177/153537020322801006

[29] Itoh Y , Oishi R , Saeki K . Feeding‐induced increase in the extracellular concentration of histamine in rat hypothalamus as measured by in vivo microdialysis. Neurosci Lett. 1991;125:235‐237. 1881601 10.1016/0304-3940(91)90037-t

[30] Ookuma K , Yoshimatsu H , Sakata T , Fujimoto K , Fukagawa F . Hypothalamic sites of neuronal histamine action on food intake by rats. Brain Res. 1989;490:268‐275. 2765863 10.1016/0006-8993(89)90244-8

[31] Gao F , Luo N , Thumboo J , Fones C , Li SC , Cheung YB . Does the 12‐item General Health Questionnaire contain multiple factors and do we need them? Health Qual Life Outcomes. 2004;2:63. 15538951 10.1186/1477-7525-2-63 PMC534792

[32] Herhaus B , Passler S , Petrowski K . Stress‐related laboratory eating behavior in adults with obesity and healthy weight. Physiol Behav. 2018;196:150‐157. 30170169 10.1016/j.physbeh.2018.08.018

[33] Bergdahl M , Bergdahl J . Low unstimulated salivary flow and subjective oral dryness: association with medication, anxiety, depression, and stress. J Dent Res. 2000;79:1652‐1658. 11023259 10.1177/00220345000790090301

[34] Ben‐Aryeh H , Miron D , Szargel R , Gutman D . Whole‐saliva secretion rates in old and young healthy subjects. J Dent Res. 1984;63:1147‐1148. 6589278 10.1177/00220345840630091001

[35] Heft MW , Baum BJ . Unstimulated and stimulated parotid salivary flow rate in individuals of different ages. J Dent Res. 1984;63:1182‐1185. 6592197 10.1177/00220345840630100101

